# Dehydrocostus Lactone Inhibits Microglia‐Mediated Neuroinflammation by Targeting CYP2A6 to Improve Ischemic Brain Injury

**DOI:** 10.1111/cns.70502

**Published:** 2025-07-05

**Authors:** Xin Shu, Xinxin Zou, Jingxuan Zhang, Xu Fang, Xinyu Wang, Hui Wu, Xuan He, Dujuan Sha

**Affiliations:** ^1^ Department of General Practice Nanjing Drum Tower Hospital, Affiliated Hospital of Medical School, Nanjing University Nanjing China; ^2^ The State Key Laboratory of Pharmaceutical Biotechnology, Nanjing University Nanjing China; ^3^ Department of Neurology Nanjing Drum Tower Hospital, Affiliated Hospital of Medical School, Nanjing University Nanjing China; ^4^ Department of General Practice Nanjing Drum Tower Hospital Clinical College of Nanjing University of Chinese Medicine Nanjing China

**Keywords:** CYP2A6, dehydrocostus lactone, ischemic stroke, microglia, neuroinflammation

## Abstract

**Background:**

Neuroinflammation is an important factor in ischemic stroke. Dehydrocostus lactone (DHC) plays an anti‐inflammatory role in certain diseases. However, the role of DHC in neuroinflammation after ischemic stroke remains unclear.

**Methods:**

DHC was administered to lipopolysaccharide (LPS)‐treated BV2 cells and a middle cerebral artery occlusion (MCAO) model to detect the levels of inflammatory factors using quantitative real‐time PCR, western blotting, and behavioral tests. Morphological changes in microglia were observed using immunofluorescence. The Swiss Target Prediction database was used to predict the target of DHC. Finally, a specific inhibitor of the target protein was used to investigate its potential synergistic role in neuroinflammation, both with and without being combined with DHC.

**Results:**

The expression of inflammation‐related factors both in vivo and in vitro was improved by DHC, and the neurological deficits in mice after MCAO were improved in the DHC administration group. In addition, the Swiss Target Prediction showed that CYP2A6 was a target of DHC. Specifically, the combination of DHC with the CYP2A6 inhibitor showed that DHC exerts anti‐inflammatory effects in a CYP2A6‐dependent manner.

**Conclusion:**

Mechanistically, DHC inhibited neuroinflammation by binding to the target CYP2A6. Our study suggests that DHC is a promising new strategy for treating ischemic stroke.

## Graphical abstract

Dehydrocostus lactone (DHC) inhibits microglia‐mediated neuroinflammation by targeting CYP2A6, reducing the release of pro‐inflammatory factors and improving nerve function impairment, thereby alleviating ischemic brain injury.

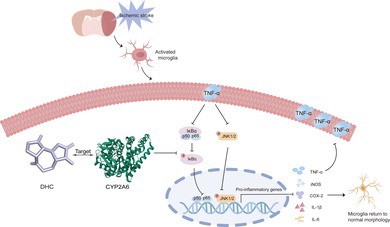

## 1 Introduction

Ischemic stroke, with its high incidence and rates of disability and mortality, is the second leading cause of life‐threatening health issues globally [1]. It occurs when the brain vessels are blocked, leading to hypoxia and ischemia and subsequently causing cell death in the central nervous system [2]. The onset and progression of ischemic stroke involve a series of pathophysiological mechanisms such as glutamate toxicity, brain–blood barrier disruption, oxidative stress, and neuroinflammation, which aggravate the brain injury [3, 4, 5]. Currently, intravenous thrombolysis with recombinant tissue plasminogen activator (rt‐PA) and vascular interventional thrombolysis are used to treat ischemic stroke [6]. However, owing to the limited time window and poor prognosis of secondary brain injury after reperfusion, it is imperative to develop drugs to extend the time window and reduce complications.

Microglia are the resident immune cells in the brain that monitor brain activity and maintain brain homeostasis [7]. When injury and stimulation occur in the brain, microglia are rapidly activated, migrate to the damaged area, and undergo phenotypic changes [8]. The number of microglia in the ischemic penumbra gradually increases and peaks 3 days later. The activated microglia can release large amounts of pro‐inflammatory factors such as TNF‐α, IL‐6, and IL‐1β to trigger neuroinflammation, aggravating secondary brain injury [9]. These pro‐inflammatory factors cause endothelial cell apoptosis, disrupt the blood–brain barrier, and lead to extensive neutrophil infiltration [10, 11, 12]. Therefore, inhibiting the release of inflammatory factors by microglia and exploring their potential mechanisms are potential therapeutic strategies to reduce neuroinflammation and promote brain tissue repair.

Dehydrocostus lactone (DHC), a sesquiterpene lactone isolated from the root of *Saussurea costus* , is a major bioactive component of several traditional Chinese medicines and has been previously used in gastrointestinal diseases [13, 14]. Numerous studies have shown that DHC possesses anti‐tumor and anti‐inflammatory properties [15, 16]. Previous studies have found that DHC can inhibit the allergic airway inflammation and reduce damage from ulcerative colitis by balancing the gut microbiota [17, 18]. Additionally, DHC suppressed macrophage activation to alleviate lung injury [19] and could be used to treat sepsis by inhibiting the release of inflammatory factors [20]. However, the role of DHC in the nervous system, particularly during ischemic brain injury, remains unclear.

Our research found that DHC can inhibit the activation of microglia by binding to the target CYP2A6 and reduce the generation of pro‐inflammatory factors through the TNF pathway, thus mitigating neurological functional impairment. Overall, DHC is a candidate drug for the treatment of ischemic brain injury.

## 2 Methods

### 2.1 Animals

The 8‐week‐old male C57BL/6J mice used in this study were purchased from GemPharmatch Co. Ltd. (Nanjing, Jiangsu, China) and housed in a specific pathogen‐free facility with controlled temperature and humidity, and a 12‐h light/dark cycle, with free access to food and water. All animal experiments were conducted in strict accordance with the guidelines and approved by the Ethics Committee. The decision to use only male mice was guided by several considerations. Hormone levels in female mice, such as estrogen, fluctuate throughout the estrous cycle and may influence neurological recovery following ischemic brain injury. Previous studies have demonstrated that estrogen exerts protective effects against ischemic stroke by improving cerebral perfusion, reducing oxidative stress, and attenuating inflammatory responses [21, 22, 23]. To avoid variability introduced by these hormonal fluctuations and ensure consistency in experimental conditions, we opted to use only male mice in this initial study. This choice aligns with numerous prior studies on ischemic stroke pathology, most of which utilized male mice [24, 25], thus enabling direct comparisons between our findings and existing literature. As this study represents our initial investigation into the mechanisms underlying the effects of DHC on ischemic brain injury, selecting male mice allowed us to effectively validate our core hypotheses without confounding factors.

### 2.2 Transient Middle Cerebral Artery Occlusion Model and Drug Treatment

Mice were anesthetized with 2.5% avertin, and the right common carotid artery and its external and internal branches were isolated through a midline cervical incision. A filament was inserted into the external carotid artery to occlude the middle cerebral artery. One hour later, the filament was removed to allow for reperfusion. The mice were randomly divided into the sham, MCAO, and DHC groups. The DHC group received intraperitoneal injections of 10 mg/kg DHC (CAS: 477‐43‐0, Aladdin, Shanghai, China) 30 min after model creation and daily on the second and third days. The MCAO group received intraperitoneal injections of DMSO at the same time points for 3 days. After drug administration, behavioral and subsequent experiments were conducted.

### 2.3 Behavioral Tests

All mice underwent behavioral training twice daily for three consecutive days immediately preceding MCAO induction. Baseline performance was assessed on the final day of training, one day before MCAO. Mice unable to reliably perform the behavioral tasks after training sessions were excluded from subsequent experiments. Specifically, mice that consistently failed to maintain balance on the rotarod or repeatedly displayed a high error rate in the foot fault test were excluded. This ensured that all animals included in the study had stable and consistent baseline behavioral performance prior to MCAO induction. The rotarod test was used to assess balance and sensorimotor coordination. The mice were placed on a rotating rod, with the speed adjusted to 10, 20, and 30 rpm, each for 5 min, with a 5‐min interval between speeds. Subsequent training sessions were conducted at 20, 30, and 40 rpm for 5 min. The time taken to fall off the rod at 40 rpm was recorded as the baseline, and the same was recorded on the test day. The foot fault test was used to assess motor function. The mice were placed on a grid to walk freely, and the number of lost steps in the left upper limb within 50 steps was counted as the baseline, with the same values recorded on the test day. The grip strength test was used to assess muscle function. The mice were lifted by their tail to grasp a horizontal bar with their forepaws, and the grip strength was recorded multiple times. The modified neurological severity score (mNSS) was used to evaluate the severity of neurological deficits, including motor, sensory, and reflex functions. The sum of these values was recorded on the test day. The mice were lifted to observe the bending of their forelimbs and hind limbs, as well as walking on flat ground. The mice were placed on a balance beam to observe their falling behavior.

### 2.4 TTC Staining

The mice were sacrificed by cervical dislocation, and their brains were removed and sliced using a mold. The slices were then immediately stained with 2% 2,3,5‐Triphenyltetrazolium chloride (TTC) staining solution (T8877, Sigma‐Aldrich, St. Louis, MO, USA). After the color change, the TTC solution was removed, and the slices were photographed. Infarct area was calculated using ImageJ software (National Institutes of Health, Bethesda, MD, USA).

### 2.5 Cell Culture

Primary microglial cells were isolated from the brains of newborn mice. The heads of the newborn mice were cut off, and the whole brains were separated and digested with trypsin (T2600000, Sigma‐Aldrich, St. Louis, MO, USA) at 37°C for 10 min. The trypsin was then discarded, and the cells were resuspended in medium containing 10% serum (BC‐SE‐FBS01, Biochannel, Nanjing, China) and 1% Penicillin–Streptomycin (BC‐CE‐007, Biochannel, Nanjing, China) and cultured at 37°C. The medium was changed at 48 h and 1 week after plating. Cells were collected on days 10 and 13 for subsequent experiments. BV2 cells were cultured in medium supplemented with 10% serum and 1% Penicillin–Streptomycin at 37°C.

### 2.6 CCK‐8 Assay

BV2 cells were plated overnight at a density of 8000 cells/well in 96‐well plates. The cells were treated with DMSO or DHC for 24 h, and the medium was removed. CCK‐8 solution was prepared according to the instructions of CCK‐8 Cell Counting Kit (A311‐01, Vazyme, Nanjing, China), and 100 μL of the solution was added to each well. The plates were incubated at 37°C for 1 h, and the absorbance was measured at 450 nm using a microplate reader. Cell viability was calculated as (experimental group—blank group)/(control group—blank group) × 100%. Cells pretreated with DHC for 2 h were stimulated with 500 ng/mL LPS for 24 h, and then cell viability was determined.

### 2.7 LDH Assay

BV2 cells were treated as described above. LDH assay was performed according to the LDH kit instructions (11644793001; Roche Diagnostics GmbH, Mannheim, Germany). In general, the maximum lysis group was pretreated with lysis buffer for 15 min to ensure complete cell death and served as a control. One hundred microliters of dye were added to each well and incubated at room temperature for 30 min, followed by the addition of stop solution, and the absorbance was measured at 490 nm. The cell death rate was calculated as follows: (experimental group—background group)/(maximum lysis group—background group) × 100.

### 2.8 Enzyme‐Linked Immunosorbent Assay

After collecting the supernatants from each group, IL‐6, TNF‐α, and IL‐1β proteins were detected according to the instructions provided (ABclonal, Wuhan, China).

### 2.9 Whole Transcriptome Sequencing

Whole transcriptome sequencing was conducted by Shanghai Majorbio Bio‐Pharm Technology Co. Ltd. Briefly, total RNA was extracted and the library was enriched. Quality control of the sequencing data was carried out using the fastp software to eliminate low‐quality sequences. The software HiSat2 was employed for alignment with the reference genome, and RSEM was used to quantify gene expression levels. DESeq2 was used to analyze gene differences. The criteria for significantly different genes were FDR < 0.05, and |log2FC| > 1. Kyoto Encyclopedia of Genes and Genomes (KEGG) pathway enrichment analysis was performed using the Python software package and calculated using the Fisher's test.

### 2.10 Immunofluorescence

After anesthetizing mice with 2.5% avertin, PBS and 4% paraformaldehyde were perfused through the right atrial auricular until the liver turned white. Then the mice were sacrificed by cervical dislocation, and their brains were removed. The detached brains were fixed overnight with 4% paraformaldehyde and then sequentially dehydrated in 15% and 25% sucrose solutions for 24 h each. Brain slices of 20 μm thickness were cut by CryoStar NX50 (Thermo Fisher Scientific, Waltham, MA, USA) for further experiments. Primary microglial cells were plated in confocal dishes and treated with DHC for 2 h, followed by stimulation with 100 ng/mL LPS ( *Escherichia coli* 055: B5, Sigma‐Aldrich, St. Louis, MO, USA) for 1.5 h. The cells were fixed with 4% paraformaldehyde for 20 min and rinsed with PBS × 10 min each. Cells were permeabilized with 0.25% Triton X‐100 and blocked with 5% BSA for 1 h, followed by incubation with primary antibodies anti‐Iba1 (Goat, Abcam, 1:500), anti‐TMEM119 (Rabbit, Abcam, 1:50), and anti‐CD86 (Rabbit, Abcam, 1:100) overnight at 4°C. The next day, after rinsing for 3 × 10 min with PBS, secondary antibodies were applied and incubated at room temperature in the dark for 1 h. After washing with PBS, the nuclei were stained with DAPI (ab104139, Abcam, Cambridge, MA, USA), and imaging was performed using a STELLARIS 5 Confocal Microscope (Leica Microsystems CMS GmbH, Mannheim, Germany) at 20× magnification.

### 2.11 qRT‐PCR

The BV2 cells were plated at a density of 80,000 cells/well in 24‐well plates. Cells were treated with DHC for 2 h, followed by stimulation with 500 ng/mL LPS for 3 h or replaced with sugar‐free medium and placed in a container containing 95% N_2_ and 5% CO_2_ for oxygen–glucose deprivation for 1 h, and then replaced with complete medium for reoxygenation for 3 h. Total RNA was extracted using Vezol Reagent (R411, Vazyme, Nanjing, China) and reverse‐transcribed into cDNA. Real‐time quantitative PCR was performed using the ChamQ Universal SYBR qPCR Master Mix (Q711‐02, Vazyme, Nanjing, China), and the expression levels were obtained using the QuantStudio 5 Real‐Time PCR System (Applied Biosystems, Foster City, CA, USA). The results were normalized to the GAPDH expression levels. The primer sequences used were as follows:

iNOS Forward TCCAGGATGAGGACATGAGCAC

Reverse GAACGTCACACACCAGCAGGTTA

COX‐2 Forward TGAGCAACTATTCCAAACCAGC

Reverse GCACGTAGTCTTCGATCACTATC

IL‐1β Forward CCATCCTCTGTGACTCATGGG

Reverse TCAGCTCATATGGGTCCGAC

IL‐6 Forward GACAAAGCCAGAGTCCTTCAGAGAG

Reverse CTAGGTTTGCCGAGTAGATCTC

TNF‐α Forward CCACCACGCTCTTCTGTCTA

Reverse GATCTGAGTGTGAGGGTCTGG

GAPDH Forward AGGTCGGTGTGAACGGATTTG

Reverse TGTAGACCATGTAGTTGAGGTCA

### 2.12 Western Blotting

Proteins from the ischemic penumbra tissue of the mouse cerebral cortex and cells were extracted using radioimmunoprecipitation assay (RIPA) lysis buffer (P0013K; Beyotime, Shanghai, China) containing protease and phosphatase inhibitors. After the protein was lysed on ice for half an hour, it was centrifuged at 12,000 rpm for 20 min. The supernatant was then taken and quantified using a BCA Protein Quantification Kit (E112, Vazyme, Nanjing, China). Denatured proteins were separated by electrophoresis using 10% glue and transferred onto a PVDF membrane. Membranes were blocked with 5% skim milk for 1 h and incubated with primary antibodies anti‐iNOS (Rabbit, Abways, 1:1000), anti‐COX‐2 (Rabbit, Bioworld, 1:1000), anti‐TNF‐α (Mouse, Abcam, 1:1000), anti‐phospho‐p65 (Rabbit, Cell signaling technology, 1:1000), anti‐p65 (Rabbit, Cell signaling technology,1:1000), anti‐phospho‐IκBα (Rabbit, Cell signaling technology, 1:1000), anti‐IκBα (Rabbit, Abways, 1:1000), anti‐phospho‐JNK (Rabbit, Cell signaling technology, 1:1000), anti‐JNK (Mouse, Proteintech, 1:3000), and anti‐GAPDH (Rabbit, ABclonal, 1:300000) overnight at 4°C. After washing thrice with PBST, the membranes were incubated with secondary antibodies at room temperature for 1 h. After three more washes, ECL reagent (BL520A, Biosharp, Beijing, China) was added, and the blots were developed using an Automatic Chemiluminescence Imaging Analysis System (Tanon, Shanghai, China). Bands were quantified using ImageJ software (National Institutes of Health, Bethesda, MD, USA).

### 2.13 Statistical Analysis

All Statistical analyses were performed using GraphPad Prism version 9.0 (GraphPad Software, La Jolla, CA, USA). Data were presented as mean ± SEM. All data distributions were determined by a normality test, followed by Shapiro–Wilk test. Significant differences between two groups were determined by unpaired Student's *t*‐test, whereas significant differences among multiple groups were determined by one‐way ANOVA, followed by Bonferroni's post hoc test. Statistical significance was considered when *p* < 0.05.

## 3 Results

### 3.1 DHC Exhibits a Safe Concentration Range for Microglial Cells

**FIGURE 1:**
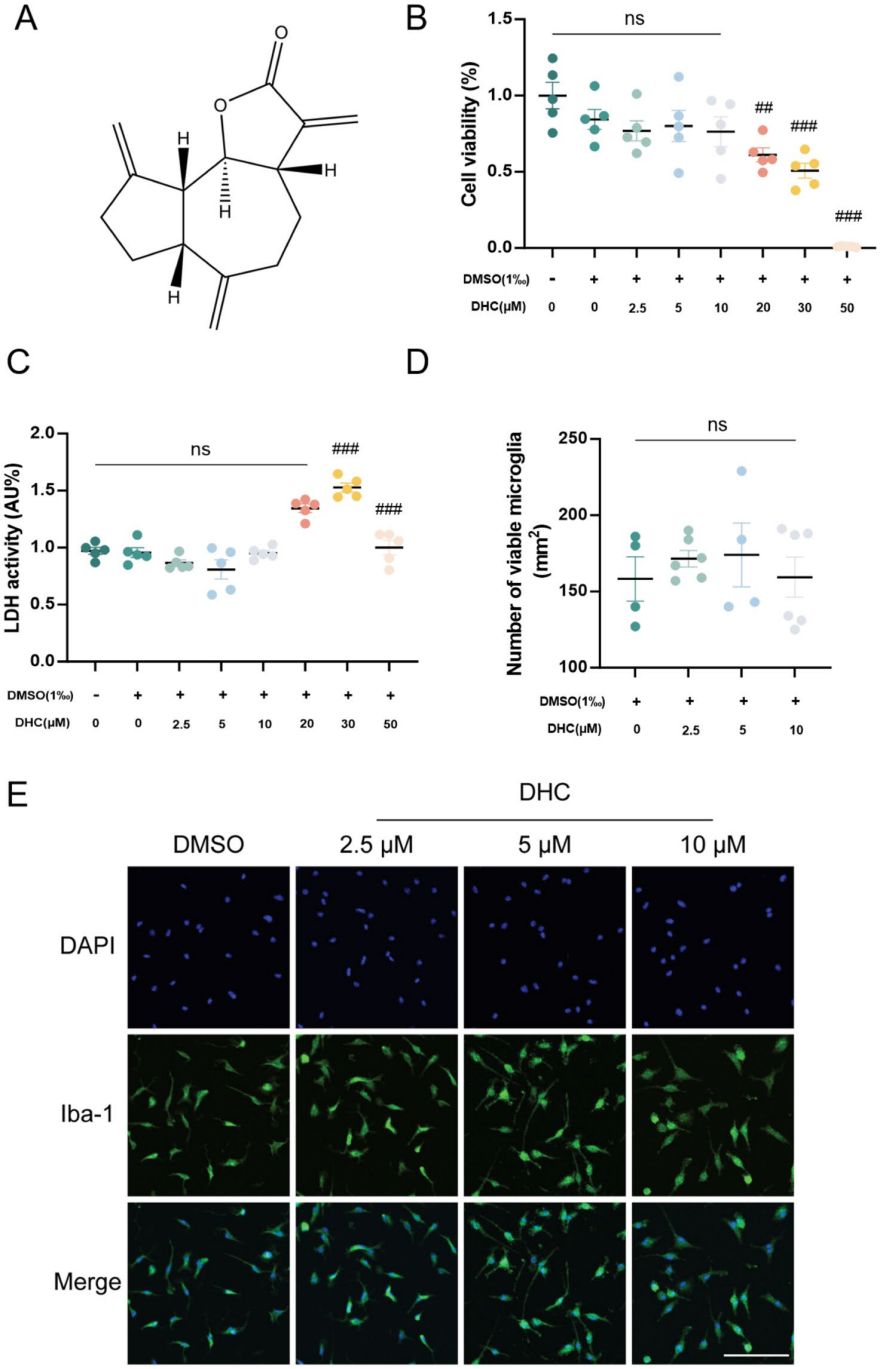
DHC is a herbal extract with a safe microglia concentration range. (A) Chemical structure of DHC. (B, C) Primary microglia cells were treated with DHC in different doses (0, 2.5, 5, 10, 20, 30, 50 μM), then CCK‐8 assay and LDH cytotoxicity assay was added to detect cell viability 24 h later, respectively. (D–E) Immunofluorescence staining was used to detect the number of viable primary microglia (bar = 100 μm). Values are presented as means ± SEM. ###*p* < 0.001 compared with control. Comparison was from at least three independent experiments.

DHC is a lipophilic compound extracted from snow lotus and is composed of lactone and aromatic rings (Figure 1A). To determine the safe range in BV2 cells, we used CCK‐8 and LDH assay kits to test the viability and death rates at various concentrations of DHC. Compared to the control group, a concentration of DHC at 2.5, 5, and 10 μM had no obvious effect on the cell viability and death rates, while at 30 and 50 μM a significant increase in cell death rates was reported (Figure 1B,C). Furthermore, immunofluorescence staining was used to confirm the effective concentration of DHC in primary microglial cells. We did not find any significant difference in the number of Iba‐1‐positive microglial cells with any tested concentration (Figure 1D,E). In summary, DHC at concentrations of 2.5, 5, and 10 μM showed no toxicity to microglia, which were chosen in subsequent experiments. Additional assays under LPS stimulation further demonstrated significant cytotoxicity at or above 25 μM, thereby establishing 10 μM as the safe upper therapeutic limit (Figure S1).

### 3.2 DHC Suppresses the Expression of LPS‐Induced Pro‐Inflammatory Factors in BV‐2 Microglial Cells

**FIGURE 2:**
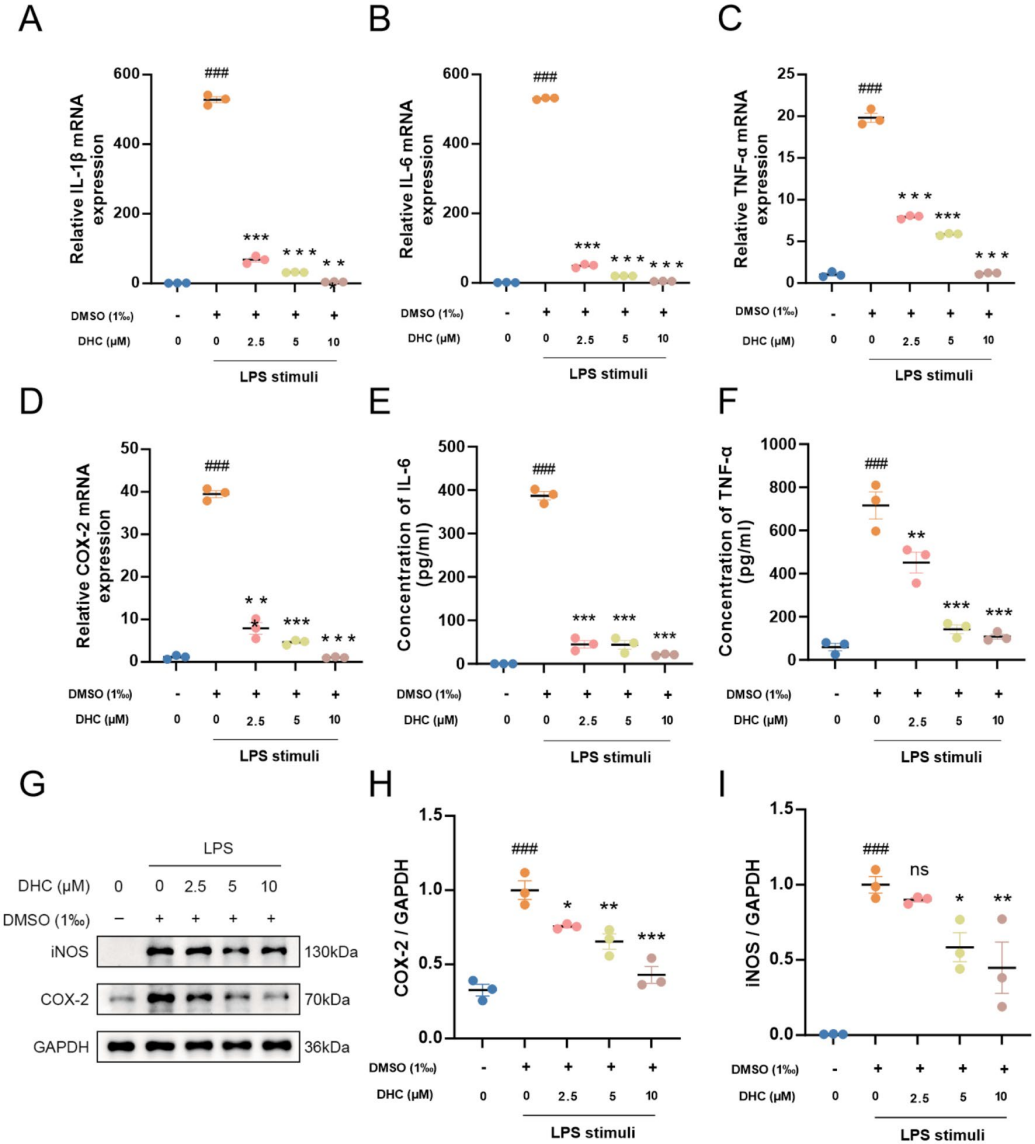
DHC suppresses the expression of LPS‐induced pro‐inflammatory factors in BV‐2 microglial cells. BV2 cells were stimulated with LPS (500 ng/mL, 3 h for mRNA and 24 h for protein levels detection) before DHC administration in different concentrations. (A–D) Total RNA was isolated and the mRNA levels of IL‐1β, IL‐6, TNF‐a and COX‐2 were quantified by RT‐qPCR. (E, F) The cell lysates and the supernatant were collected after LPS‐used for 24 h. The protein in the supernatant like IL‐6 (E) and TNF‐a (F) were detected using ELISAs (*n* = 3). (G–I) Westernblot were used to measure the protein levels of iNOS and COX‐2 in cell lysates, with GAPDH as a loading control. The ratio of COX‐2/GAPDH(H) and iNOS/GAPDH (I) were analyzed by ImageJ software. The values are shown as means ± SEM, *n* = 3. ###*p* < 0.001 compared with control groups. *p < 0.05, **p < 0.01, ***p < 0.001 compared with LPS‐treated groups.DHC suppresses the expression of LPS‐induced pro‐inflammatory factors in BV‐2 microglial cells. BV2 cells were stimulated with LPS (500 ng/mL, 3 h for mRNA and 24 h for protein levels detection) before DHC administration in different concentrations. (A–D) Total RNA was isolated and the mRNA levels of IL‐1β, IL‐6, TNF‐a and COX‐2 were quantified by RT‐qPCR. (E, F) The cell lysates and the supernatant were collected after LPS‐used for 24 h. The protein in the supernatant like IL‐6 (E) and TNF‐a (F) were detected using ELISAs (*n* = 3). (G–I) Westernblot were used to measure the protein levels of iNOS and COX‐2 in cell lysates, with GAPDH as a loading control. The ratio of COX‐2/GAPDH(H) and iNOS/GAPDH (I) were analyzed by ImageJ software. The values are shown as means ± SEM, *n* = 3. ###*p* < 0.001 compared with control groups. *p < 0.05, **p < 0.01, ***p < 0.001 compared with LPS‐treated groups.

In order to explore the inhibition effect of DHC on inflammatory responses, mRNA levels of pro‐inflammatory factors IL‐1β, IL‐6, TNF‐α, and COX‐2 were detected by qRT‐PCR. As shown in Figure 2A–D, the levels of pro‐inflammatory factors were significantly elevated in the LPS group compared to the control group, and treatment with DHC significantly inhibited the release of these cytokines in a dose‐dependent manner, with higher concentrations exhibiting greater inhibitory effects. The protein levels of cytokines in cell lysates and supernatants detected by western blotting and ELISA showed a significant increase in the LPS‐treated group compared with that in the control group, but significantly decreased in the DHC‐treated group, consistent with findings related to the mRNA (Figure 2E–I). Overall, DHC effectively inhibited the release of pro‐inflammatory factors in activated BV2 cells.

### 3.3 DHC Alleviates Microglia‐Mediated Neuroinflammation After Experimental Stroke

**FIGURE 3:**
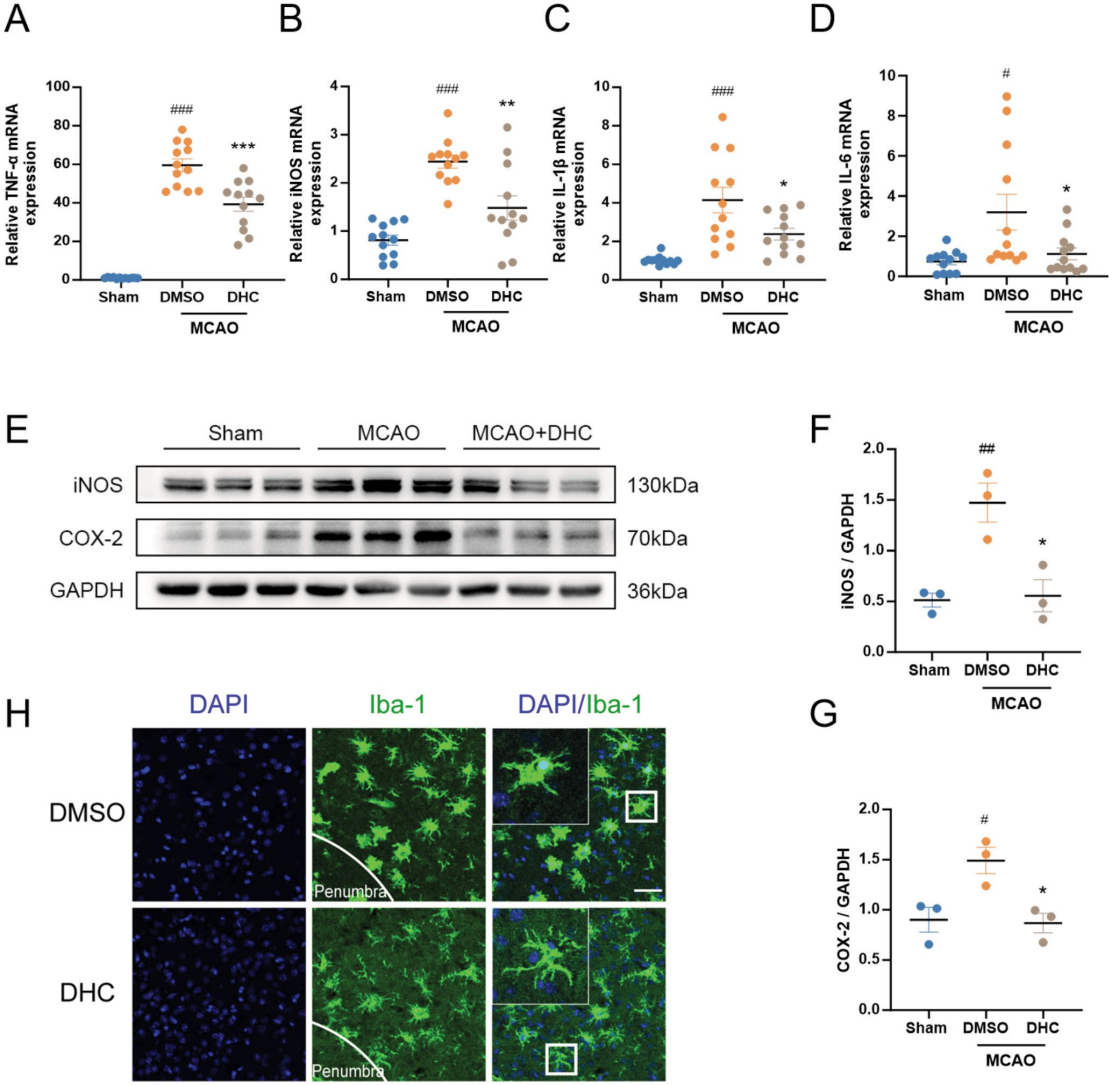
DHC alleviates post‐stroke microglia‐mediated neuroinflammation after experimental stroke. Mice subjected to tMCAO were administered with/without DHC (10 mg/Kg, once a day for 3 days) via i.p. injection. Then penumbra of brain tissues after ischemia from tMCAO mice was collected for RT‐qPCR or western blot. (A–D) Total RNA of penumbra tissue was extracted and the mRNA levels of pro‐inflammatory factors like IL‐1β, IL‐6, TNF‐a and iNOS were assessed by RT‐qPCR (*n* = 12). (E–G) Westernblot was used to examine protein levels of iNOS, COX‐2 and GAPDH in penumbra tissue. The quantifications of relative band intensities were determined by densitometry (*n* = 3). (H) Brain tissue slices from mice above were collected and stained with microglia biomarker Iba‐1 (green) and nucleus DAPI (blue), Scale bars = 20 μm. Values are expressed as the mean ± SEM. #*p* < 0.05, ##*p* < 0.01, ###*p* < 0.001versus sham groups. *p < 0.05, **p < 0.01, ***p < 0.001 compared with DMSO‐administrated groups.

Microglia‐mediated neuroinflammation is a key factor in stroke outcomes. We further investigated the role of DHC in post‐stroke pro‐inflammatory responses. The mice were divided into three groups (Sham, DMSO‐treated, and DHC‐treated) and subjected to MCAO surgery, with the exception of the sham group. Penumbra tissues and brain slices from mice in each group were collected 3 days after MCAO. Our results showed that the mRNA levels of pro‐inflammatory factors such as TNF‐α, iNOS, IL‐1β, and IL‐6 were significantly increased after MCAO surgery and obviously decreased after treatment with 10 mg/kg DHC (Figure 3A–D). Similarly, the protein levels of iNOS and COX‐2 were decreased in the DHC‐treated group compared to the DMSO‐treated group (Figure 3E–G). In addition, microglia were activated in an amoeboid‐like shape with increased cell volume and short, thick processes after ischemic stroke, which was reversed by DHC treatment (Figure 3H). Dual immunofluorescence staining further revealed a marked reduction in CD86 expression in microglia (TMEM119‐positive cells) of the DHC‐treated group compared to the transient middle cerebral artery occlusion (tMCAO) group, indicating that DHC might suppress neuroinflammation by inhibiting M1‐type microglial activation (Figure S2). Overall, DHC reduced the microglia‐mediated neuroinflammation in the penumbra following ischemic stroke.

### 3.4 DHC Improves Mice Brain Injury After Ischemic Stroke

**FIGURE 4:**
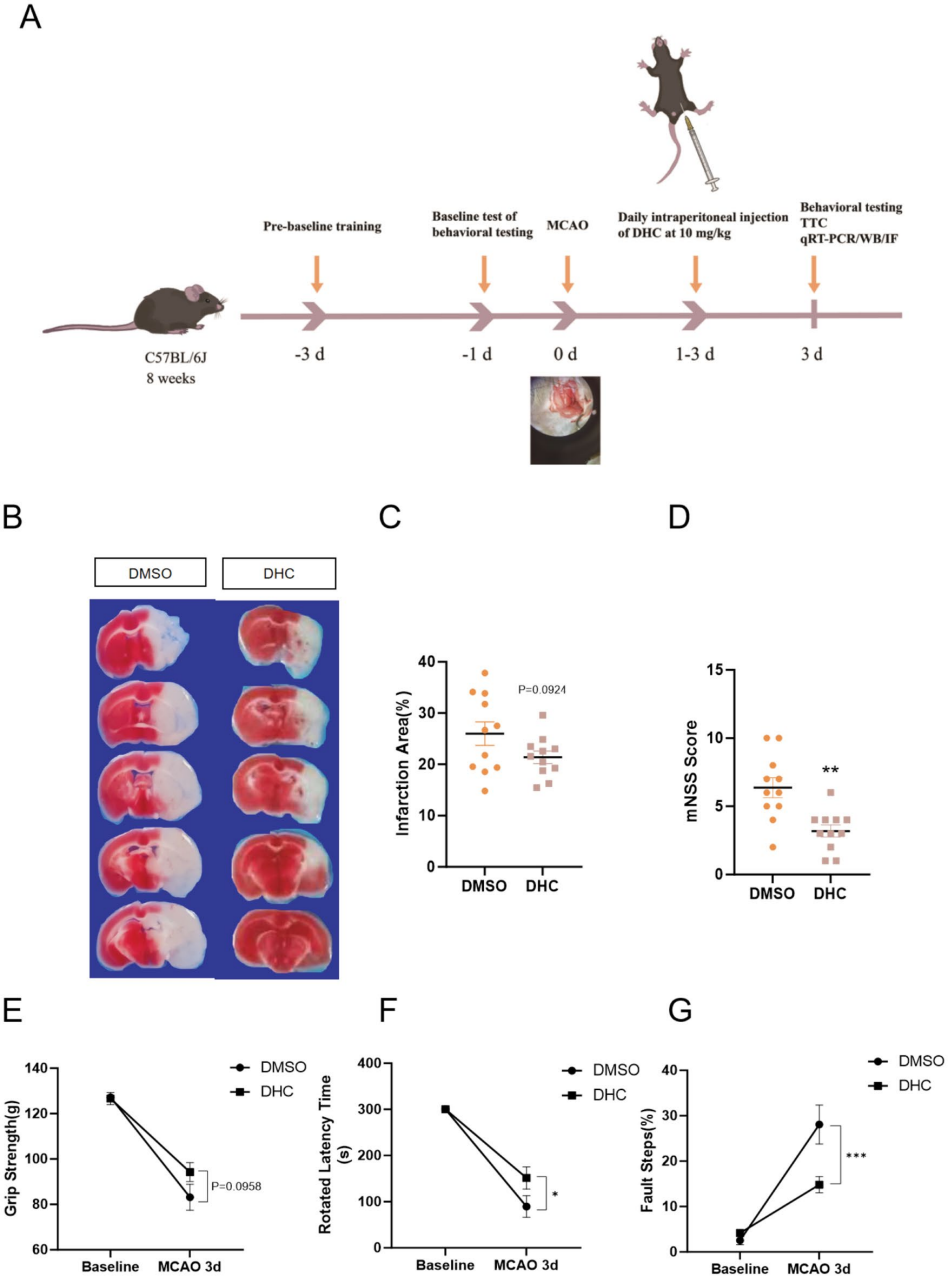
DHC improves brain injury after ischemia. Mice were subjected to tMCAO surgery and then administered with DMSO or DHC (10 mg/kg, once a day for 3 days) via i.p. injection. (A) Schematic diagram of experimental design and timelines for animal studies. (B) At the third day after tMCAO, representative images of brain sections were obtained while stained with TTC. (C) Infarction volume (*n* = 11). (D–G) Neurological deficits were evaluated by the mNSS scores (D), the grip strength (E), the rotarod test (F) and the fault steps (G). Values shown are expressed as mean ± SEM. *p < 0.05, **p < 0.01, ***p < 0.001 compared with the DMSO group.

To examine the infarct size and neurological deficits in mice, TTC and behavioral tests, including the rotarod test, foot fault, grip strength, and mNSS scores, were used in this study. The schematic timeline of animal experiments is shown in Figure 4A. Briefly, after MCAO surgery, mice were intraperitoneally administered 10 mg/kg DHC for 3 days. The mouse brains were then removed from the skull for TTC staining. The TTC staining results showed that DHC treatment reduced the infarct size after MCAO (Figure 4B,C). Behavioral tests showed that DHC treatment also alleviated the functional impairments in MCAO mice, restoring motor and balance abilities to a certain extent (Figure 4D–G).

### 3.5 DHC Suppresses Pro‐Inflammatory Response by Targeting CYP2A6, a Subtype of the Cytochrome P450 Enzyme Through TNF Pathway

**FIGURE 5:**
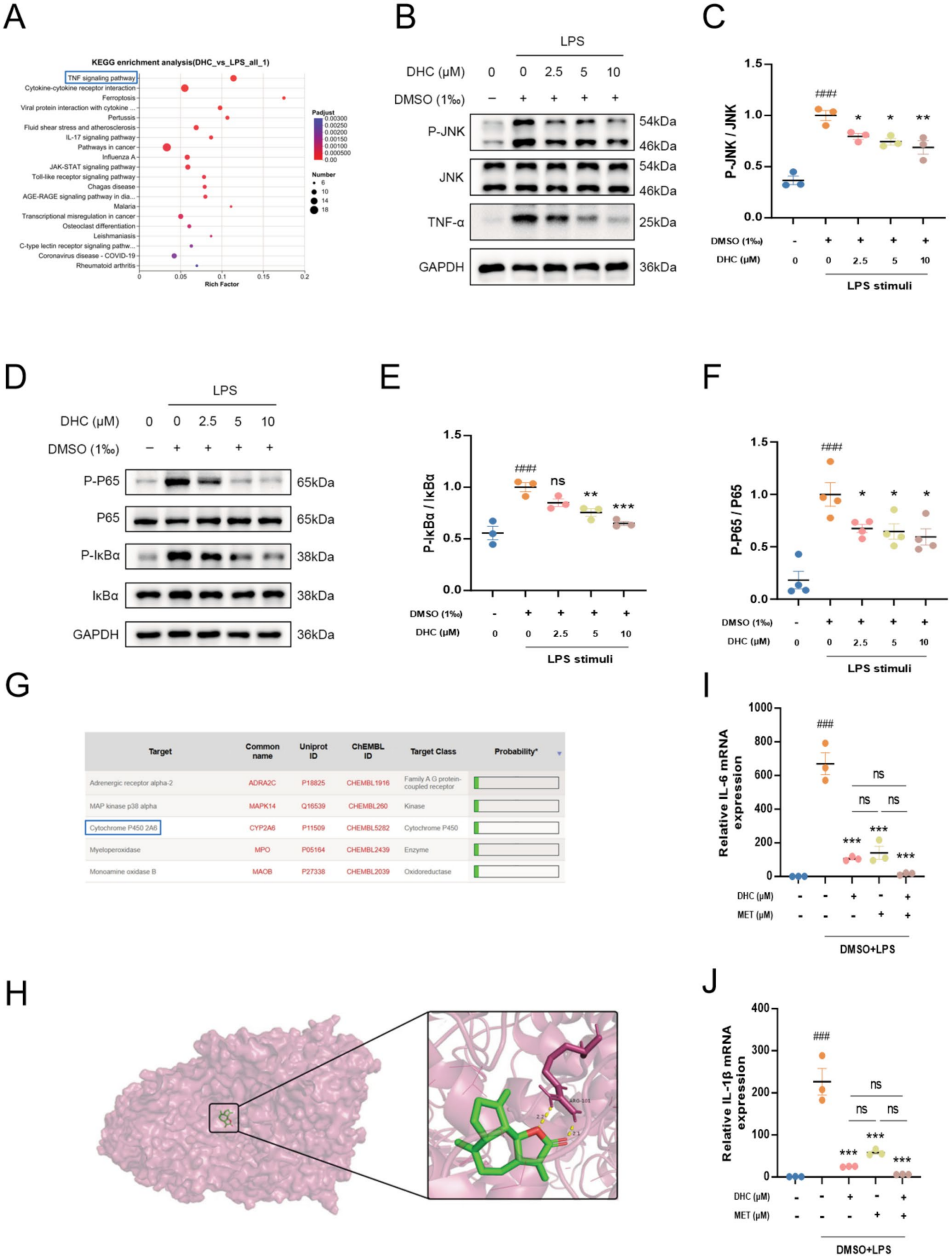
DHC suppresses pro‐inflammatory response by targeting CYP2A6, a subtype of the cytochrome P450 enzyme through TNF pathway. After being treated with LPS for 3 h, transcriptome sequencing was performed on BV‐2 cells administrated with/without DHC. (A) KEGG functional enrichment analysis. (B–F) The protein levels of TNF‐a, P‐JNK, JNK, P‐IκBα, IκBα, P‐P65, P65, and GAPDH were detected by western blot. The ratio of P‐JNK/JNK, P‐IκBα/IκBα, and P‐P65/P65 were quantified by densitometry (*n* = 3). (G) Targets of DHC were shown in the table predicted by SwissTargetPrediction database, and CYP2A6 worked as a specific protein among those. (H) Crystal structure of mouse CYP2A6 in complex with DHC was predicted by Autodock software. (I, J) BV2 cells were treated with/without DHC or Met for 2 h, then stimulated with LPS (500 ng/mL) for additional 3 h (*n* = 3 each group), total RNA was extracted and the mRNA levels of IL‐1β and IL‐6 were analyzed by RT‐qPCR. The values are shown as the mean ± SEM. ###*p* < 0.001 compared with control groups. *p < 0.05, **p < 0.01, ***p < 0.001 compared with LPS‐treated groups.DHC suppresses pro‐inflammatory response by targeting CYP2A6, a subtype of the cytochrome P450 enzyme through TNF pathway. After being treated with LPS for 3 h, transcriptome sequencing was performed on BV‐2 cells administrated with/without DHC. (A) KEGG functional enrichment analysis. (B–F) The protein levels of TNF‐a, P‐JNK, JNK, P‐IκBα, IκBα, P‐P65, P65, and GAPDH were detected by western blot. The ratio of P‐JNK/JNK, P‐IκBα/IκBα, and P‐P65/P65 were quantified by densitometry (*n* = 3). (G) Targets of DHC were shown in the table predicted by SwissTargetPrediction database, and CYP2A6 worked as a specific protein among those. (H) Crystal structure of mouse CYP2A6 in complex with DHC was predicted by Autodock software. (I, J) BV2 cells were treated with/without DHC or Met for 2 h, then stimulated with LPS (500 ng/mL) for additional 3 h (*n* = 3 each group), total RNA was extracted and the mRNA levels of IL‐1β and IL‐6 were analyzed by RT‐qPCR. The values are shown as the mean ± SEM. ###*p* < 0.001 compared with control groups. *p < 0.05, **p < 0.01, ***p < 0.001 compared with LPS‐treated groups.

After treatment with LPS, transcriptome sequencing was performed to assess the expression profile of total RNA in the lysates of BV2 cells treated with or without DHC. KEGG datasets were used to analyze differentially expressed genes (DEGs) and showed that the target proteins were mainly enriched in the TNF signaling pathway (Figure 5A). Sustained activation of the TNF signaling pathway has been verified in the pathogenesis of a series of human diseases, including diabetes, inflammatory bowel diseases, and autoimmune diseases, providing a new field of agents for the treatment of ailments [26]. The binding of TNF to TNF receptors could ultimately result in the activation of two major inflammatory pathways, JNK and nuclear factor B κB (NF‐κB) [27]. Therefore, we validated the expression of the TNF, JNK, and NF‐κB pathway‐related proteins, namely TNF‐α, JNK, p65, and IκBα. Our results showed that LPS treatment upregulated the expression level of TNF‐α and the phosphorylation levels of JNK, p65, and IκBα, which were significantly downregulated in the DHC‐treatment group (Figure 5B–F). In addition, CYP2A6, a subtype of the cytochrome P450 enzyme predicted to be the most likely target of DHC through the SwissTargetPrediction website, was mimicked as a substrate of DHC in legend‐protein interactions (the binding energy was −8.6 kcal/mol) through hydrogen bonds using Autodock software (Figure 5G,H). To verify the inhibition of this target in neuroinflammation, Methoxsalen, a CYP2A6‐specific selective inhibitor, was added to detect the mRNA levels of pro‐inflammatory factors. As expected, a significant downregulation in the release of pro‐inflammatory factors was observed in the Met‐treated group compared to that in the LPS‐stimulated group, and no differences were observed among those administered DHC, Met, or both (Figure 5I,J). Similar results were obtained under OGD conditions, where Methoxsalen significantly inhibited pro‐inflammatory cytokine expression without additional effects from combined DHC and Met treatment (Figure S3). These data indicate that DHC exerts an effect on neuroinflammatory inhibition by suppressing the activation of the TNF pathway via binding to CYP2A6.

## 4 Discussion

In this study, we discovered that DHC could effectively inhibit the levels of pro‐inflammatory factors released by activated microglia in a CYP2A6‐dependent manner and restore the neurological function in experimental mice, emphasizing the important role of DHC in alleviating neuroinflammation and ischemic brain damage.

The resident immune cells of the brain, representing 5%–20% of the glial population, are microglia [28]. Microglia play a vital role in maintaining homeostasis within the CNS nervous tissue by actively sensing and eliminating metabolic byproducts, foreign substances, and cellular debris [29]. They also participate in immune responses, synaptogenesis, neurogenesis, and neurotrophic support, thereby playing a critical role in the organism's defense mechanisms and tissue repair processes [30]. As key mediators of brain inflammation, microglia are central to the pathology of conditions such as ischemic brain injury and inflammatory neurodegenerative diseases [31]. DHC, a bioactive sesquiterpene lactone, is the main active compound extracted from the root of Saussurea lappa, a widely utilized traditional herbal medicine in China. Previous studies have extensively explored that DHC displays multiple biological activities, such as anti‐inflammatory, anti‐oxidant, and anti‐cancer [13, 32]. In this study, we found that DHC exhibited no cytotoxicity to microglial cells at concentrations up to 10 μM in vitro. However, concentrations exceeding 30 μM induced a significant increase in microglial cell death.

Neuroinflammation is characterized by an inflammatory response within the central nervous system, driven by the production of cytokines, chemokines, and inflammatory enzymes. Microglia are a specialized type of glial cell, resembling macrophages, and function as the primary immune cells in the brain and spinal cord [33]. Lipopolysaccharide (LPS), commonly known as endotoxin, is a principal component of the outer membrane of Gram‐negative bacteria that triggers a strong inflammatory response. Its application as a potent pro‐inflammatory agent serves as a well‐established model for investigating inflammation in both in vivo and in vitro studies [34]. Studies have shown that microglia are activated and release numerous inflammatory factors upon LPS induction [35, 36]. Our results indicated that the stimulation of BV2 microglial cells with LPS resulted in a significant increase in the expression of pro‐inflammatory cytokines compared to the resting state in vitro, and DHC could reverse this phenomenon, suggesting that DHC could be used as a potential therapeutic agent for the treatment of neuroinflammation‐related diseases.

Neuroinflammation has been linked to a range of neurological disorders, including stroke, Alzheimer's disease (AD), Parkinson's disease (PD), amyotrophic lateral sclerosis, and brain cancer [30]. Stroke is the second leading cause of death and a major cause of disability worldwide [37]. Neuroinflammation is widely recognized as a key element in the pathophysiology of stroke, influencing both its acute and chronic stages [38]. The inflammatory process in stroke involves the activation of various cell types, including microglia, astrocytes, endothelial cells, and leukocytes [39]. It also triggers the release of pro‐inflammatory mediators, such as cytokines, chemokines, and adhesion molecules. This inflammatory response can exacerbate brain damage, leading to secondary injury, hindering tissue repair and recovery, and contributing to post‐stroke complications like motor and cognitive impairments. Thus, neuroinflammation plays an important role in the onset and recovery from stroke [40]. The suppression of neuroinflammation in the early stages of the disease can improve ischemic brain injury [41]. Notably, our in vivo MCAO mouse model studies revealed that DHC significantly alleviated neurological deficits and reduced brain tissue damage. Subsequent studies showed that DHC treatment significantly reduced the expression of pro‐inflammatory factors in ischemic brain tissue and reversed microglia activation, suggesting that DHC may protect against ischemic brain injury by inhibiting post‐ischemic inflammation.

Cytochrome P450 enzymes are a class of monooxygenases encoded by a superfamily of genes that promote drug metabolism and lipid synthesis [42]. They are expressed in the liver and brain [43, 44]. Studies have shown that CYP2A6 is the major cytochrome P450 enzyme in macrophages [45] and plays a key role in oxidative stress mediated by nicotine metabolism in astrocytes and microglia, while being involved in arachidonic acid [46, 47, 48]. Methoxsalen is a highly selective inhibitor of CYP2A6 that exerts its inhibitory effect by inactivating the enzyme [49]. Treating BV2 cells with 200 μM Methoxsalen resulted in a significant decrease in inflammatory cytokines, suggesting that Methoxsalen has anti‐inflammatory properties. When combined with DHC, there was no significant difference in the anti‐inflammatory effects compared to the use of either DHC alone or the inhibitor group, indicating that DHC exerts its anti‐inflammatory effects in a CYP2A6‐dependent manner.

However, our study has certain limitations. TNF‐α and TNF‐β are the two most important members of the TNF superfamily [50]. TNF‐α is mainly produced by activated microglia in the brain, while TNF‐β is produced by lymphocytes [51, 52]. Although TNF‐β is also expressed in the nervous system, there are only a few studies indicating that it may play a role in the integrity of the blood–brain barrier and the regulation of partial immune responses [53, 54]. In contrast, the inflammatory regulatory role of TNF‐α is more extensive in CNS [55]. Therefore, TNF‐α has been chosen to be studied as the regulator in this article. The optimal dose and mode of action of DHC are not clear, nor have we conducted research on drug toxicity and metabolism. In vivo, the recovery of brain function after MCAO at longer time points such as 7/14 days should be further investigated in future experiments. Additionally, a specific target has been identified in vitro, and knockdown can be performed in vivo to further clarify the role of this target in neuroinflammation. However, considering that this target also plays an important role in other nerve cells, knockdown in vivo may lead to the death of mice. Moreover, systemic inhibition of CYP2A6 could potentially induce severe toxicity, and specifically targeting CYP2A6 in microglia presents significant technical challenges due to its widespread expression across various cell types within the CNS. For these reasons, additional in vivo experiments have not yet been conducted in this study. To address these limitations, future research could employ microglia‐specific genetic manipulation approaches, such as conditional knockout mice driven by microglia‐specific promoters. These future directions will significantly enhance our understanding of the precise therapeutic mechanisms underlying the effects of DHC. Gender is a critical experimental variable in drug development and neuroscience research, which will also be incorporated into future research plans.

## 5 Conclusion

In summary, this study elucidated the anti‐inflammatory effects of DHC on ischemic brain injury and the underlying molecular mechanisms. DHC inhibits neuroinflammation following ischemic brain injury by binding to CYP2A6, providing a theoretical basis for the development of anti‐neuroinflammatory drugs and new therapeutic strategies for ischemic brain injury.

## Author Contributions

D.S. designed the experiments, reviewed the manuscript, and contributed to funding acquisition. X.S. and X.Z. performed the experiments, analyzed the data, and wrote the manuscript. J.Z., X.F., X.W., and H.W. performed the experiments and analyzed the data. X.H. conceived the experiments and edited the manuscript. All authors read and approved the final manuscript.

## Conflicts of Interest

The authors declare no conflicts of interest.

## Supporting information


**Figure S1.** CCK‐8 in BV2 microglial cells treated with various concentrations of DHC under LPS stimulation. BV2 cells were pretreated with different concentrations (0, 2.5, 5, 10, 25, 50, 100 μM) of DHC for 2 h, followed by LPS stimulation (500 ng/mL) for an additional 24 h. Cell viability was measured using the CCK‐8 assay. Data are expressed as mean ± SEM (*n* = 5). ^###^ *p* < 0.001 compared with control.
**Figure S2.** Dual immunofluorescence staining of TMEM119 (green, microglia marker) and CD86 (red, M1‐type marker) in the penumbra region of ischemic mice treated with DMSO or DHC. Nuclei were stained with DAPI (blue). Scale bar = 50 μm.
**Figure S3.** Effects of DHC and Methoxsalen (Met) on pro‐inflammatory cytokine expression under oxygen–glucose deprivation (OGD) conditions. (A, B) BV2 cells were pretreated with or without DHC or Met for 2 h, then subjected to oxygen–glucose deprivation (OGD) conditions for an additional 4 h (*n* = 3 per group). Total RNA was extracted, and the mRNA levels of IL‐6 (A) and IL‐1β (B) were quantified by RT‐qPCR. The values are shown as the mean ± SEM. ^##^ *p* < 0.01, ^###^ *p* < 0.001 compared with control groups. *p < 0.05, **p < 0.01 compared with OGD‐treated groups.

## Data Availability

The data that support the findings of this study are available from the corresponding author upon reasonable request.
