# Supplementary figures and images for: Dehydrocostus Lactone Inhibits Microglia‐Mediated Neuroinflammation by Targeting CYP2A6 to Improve Ischemic Brain Injury
Source: CNS Neurosci Ther. 2025 Jul 5;31(7):e70502. doi: 10.1111/cns.70502 (PMC12227893; doi:10.1111/cns.70502)

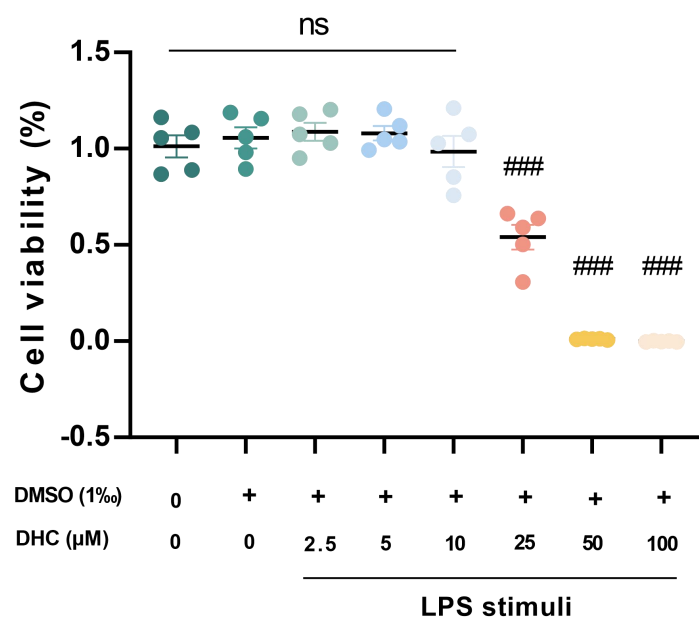

Figure S1

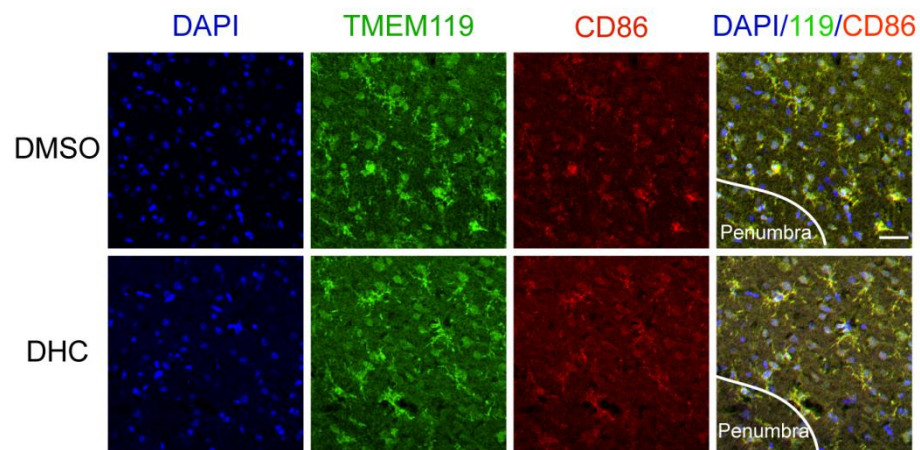

Figure S2

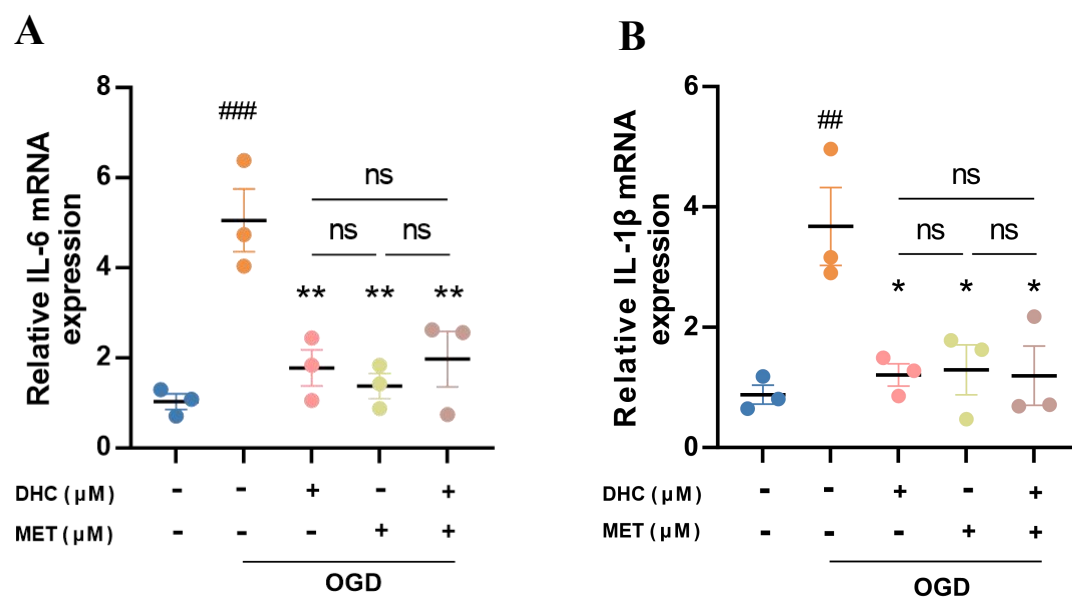

**Figure S3**

Supplement: Supplementary file 1 — Figure S1. CCK‐8 in BV2 microglial cells treated with various concentrations of DHC under LPS stimulation. BV2 cells were pretreated with different concentrations (0, 2.5, 5, 10, 25, 50, 100 μM) of DHC for 2 h, followed by LPS stimulation (500 ng/mL) for an additional 24 h. Cell viability was measured using the CCK‐8 assay. Data are expressed as mean ± SEM (n = 5). ### p < 0.001 compared with control. Figure S2. Dual immunofluorescence staining of TMEM119 (green, microglia marker) and CD86 (red, M1‐type marker) in the penumbra region of ischemic mice treated with DMSO or DHC. Nuclei were stained with DAPI (blue). Scale bar = 50 μm. Figure S3. Effects of DHC and Methoxsalen (Met) on pro‐inflammatory cytokine expression under oxygen–glucose deprivation (OGD) conditions. (A, B) BV2 cells were pretreated with or without DHC or Met for 2 h, then subjected to oxygen–glucose deprivation (OGD) conditions for an additional 4 h (n = 3 per group). Total RNA was extracted, and the mRNA levels of IL‐6 (A) and IL‐1β (B) were quantified by RT‐qPCR. The values are shown as the mean ± SEM. ## p < 0.01, ### p < 0.001 compared with control groups. *p < 0.05, **p < 0.01 compared with OGD‐treated groups. [file CNS-31-e70502-s001.pdf]
